# Identification of autophagy-related genes in intestinal ischemia-reperfusion injury and their role in immune infiltration

**DOI:** 10.3389/fphys.2025.1601968

**Published:** 2025-05-26

**Authors:** Yichen Hu, Qinghua Zou, Yanbo Sun, Weiming Li, Zhaochuan Yin, Yuanpei Zhao, Kaiwen Shi, Hongyuan Liu, Jiahui Wang

**Affiliations:** ^1^ Department of Gastrointestinal Surgery, Second Affiliated Hospital of Kunming Medical University, Kunming, Yunnan, China; ^2^ Department of Phase I Clinical Trial Ward, Yunnan Cancer Hospital, The Third Affiliated Hospital of Kunming Medical University, Kunming, Yunnan, China

**Keywords:** intestinal ischemia-reperfusion (II/R) injury, Autophagy, immune cell infiltration, bioinformatics analysis, hub genes, machine learning

## Abstract

**Background:**

Intestinal ischemia-reperfusion (II/R) injury is a serious condition characterized by high morbidity and mortality rates. Research has shown that II/R injury is closely linked to autophagy and immune dysregulation. This study aims to investigate the potential correlations between autophagy-related genes and infiltrating immune cells in II/R injury.

**Methods:**

GSE96733, GSE37013, and autophagy-related genes were obtained from the Gene Expression Omnibus (GEO) and the Human Autophagy Database, respectively. Subsequently, the biological functions of the differentially expressed genes (DEGs) were explored through DEGs analysis, Kyoto Encyclopedia of Genes and Genomes (KEGG) analysis, and Gene Ontology (GO) analysis. Using R software, human autophagy-related genes were converted to their mouse homologous autophagy-related genes (ARGs). The DEGs were then intersected with ARGs to obtain differentially expressed autophagy-related genes (DEARGs). To identify hub genes, protein-protein interaction (PPI) network analysis, Lasso regression, and random forest methods were employed. A nomogram model was constructed to assess its diagnostic value. Following this, immune infiltration analysis was performed to evaluate the potential correlation between Hub genes and immune cell infiltration. Additionally, a hub gene-related network was constructed, and potential drugs targeting hub genes for the treatment of II/R injury were predicted. Finally, the expression levels of hub genes in a mouse model of II/R injury were validated through dataset verification and quantitative real-time polymerase chain reaction (qRT-PCR).

**Results:**

Our analysis identified 11 DEARGs. Among these, 5 DEARGs (Myc, Hif1a, Zfyve1, Sqstm1, and Gabarapl1) were identified as hub genes. The nomogram model demonstrated excellent diagnostic value. Immune cell infiltration analysis indicated that these 5 hub genes are closely associated with dendritic cells and M2.Macrophage. Furthermore, the regulatory network illustrated a complex relationship between microRNAs (miRNAs) and the hub genes. Additionally, trigonelline and niacinamide were predicted as potential therapeutic agents for II/R injury. In both dataset validation and qRT-PCR validation, the four hub genes (Myc, Hif1a, Sqstm1, and Gabarapl1) showed consistency with the results of the bioinformatics analysis.

**Conclusion:**

Myc, Hif1a, Sqstm1, and Gabarapl1 have been identified as ARGs closely associated with immune infiltration in II/R injury. These hub genes may represent potential therapeutic targets for II/R injury.

## 1 Introduction

Intestinal ischemia-reperfusion (II/R) injury is a common and potentially fatal condition that arises in circumstances such as acute mesenteric ischemia, hemorrhagic or septic shock, severe burns, small intestine transplantation, and abdominal aortic surgery (Gonzalez et al., 2015). II/R injury not only induces local intestinal damage but also disrupts the intestinal mucosal barrier (Cai et al., 2025), permitting intestinal bacterial endotoxins to enter the bloodstream, which can lead to extraintestinal multiple organ dysfunction or even failure, accompanied by high morbidity and mortality rates (Pierro and Eaton, 2004). The pathophysiological mechanisms underlying II/R injury are complex and involve various factors, including oxidative stress (Jia et al., 2020), inflammatory responses, apoptosis (Cao et al., 2023), ferroptosis (Li et al., 2020), and autophagy (Chen et al., 2023). Oxidative stress is a key component in the pathogenesis of II/R injury, and the excessive production of reactive oxygen species (ROS) is one of the critical factors exacerbating II/R injury (Sasaki and Joh, 2007). Nuclear factor erythroid 2-related factor 2 (Nrf2) is a crucial transcription factor that regulates cellular antioxidant responses, playing a vital role in maintaining cellular redox balance and protecting against oxidative stress-related damage (Bae et al., 2024). Recent studies have reported that activating the Nrf2-mediated signaling pathway is one of the important mechanisms for alleviating II/R injury (Li et al., 2021). Bryostatin-1 mitigates intestinal barrier dysfunction and oxidative stress induced by II/R injury by activating the Nrf2/HO-1 signaling pathway, which supports this perspective (Liu M. et al., 2023). Apoptosis is another major contributor to cell death in II/R injury (Zu et al., 2016). By inhibiting apoptosis markers (Bax and caspase 3/9) or overexpressing anti-apoptotic markers (Bcl2), the severity of II/R injury can be reduced (Wen et al., 2019; Almoiliqy et al., 2020). Currently, certain therapeutic effects have been achieved in treating II/R injury through anti-inflammatory, antioxidant, and anti-apoptotic actions (Wang et al., 2021); however, diagnostic and treatment methods for II/R injury remain limited. Therefore, identifying key biomarkers and exploring new therapeutic strategies are crucial.

Autophagy is a highly conserved cellular process that plays a crucial role in maintaining cellular homeostasis by degrading and recycling damaged organelles, proteins, and other cellular components (Liu S. et al., 2023). Numerous diseases, particularly cancer, diabetes, heart disease, and muscle disorders, are associated with impaired autophagy (Klionsky et al., 2021). An increasing number of studies have demonstrated that mammalian target of rapamycin (mTOR), AMP activated protein kinase (AMPK), Nrf2, Beclin-1, and p62 are associated with II/R injury and represent promising therapeutic targets for this condition (Chen et al., 2020; Shen et al., 2023). As an important key regulator, mTOR affects autophagic activity and is a key target for the autophagic pathway (Zhenzhen et al., 2022). AMPK, by modulating autophagy, plays a pivotal role as an energy-sensing signaling molecule in II/R injury (Cai et al., 2022). Recent studies have shown that inflammation, oxidative stress, and apoptosis in I/R injury can be alleviated by activating AMPK/mTOR signaling and modulating autophagy (Shen et al., 2023). Nrf2 has been proven to be involved in autophagy, and research has demonstrated that enhancing autophagy through the Akt/GSK-3β/Nrf2 pathway can mitigate II/R injury (Pajares et al., 2016; Chen et al., 2020). These findings indicate that autophagy plays a crucial role in II/R injury. However, the autophagy-related genes involved in II/R injury remain largely unknown. Therefore, utilizing bioinformatics to deeply explore autophagy-related markers in II/R injury is instrumental in discovering new potential biomarkers for this condition. Furthermore, studies have shown that immune regulation plays a pivotal role in the pathogenesis of II/R injury (Hu et al., 2022; Zhang F. L. et al., 2023). Recent data demonstrates an important role for lymphocytes, particularly T cells but also B cells in II/R injury (Linfert et al., 2009). However, there have been few reports that comprehensively investigate the relationship between the expression of ARGs and immune infiltration in II/R injury, as well as the biological functions of ARGs. In recent years, non-coding RNAs, including but not limited to long non-coding RNA (lncRNA), circular RNAs (circRNA), and microRNA (miRNA), have garnered particular attention (Zhang J. et al., 2023). Research has demonstrated that the microRNA-122a inhibitor activates the EGFR-NLRP3 signaling pathway, significantly reducing pyroptosis and alleviating II/R injury, thereby highlighting the importance of miRNA in regulating II/R injury (Wang et al., 2022).

Although numerous studies have provided preliminary evidence for the regulatory role of autophagy dysregulation in II/R injury, most research has focused primarily on the biological functions of specific genes, neglecting their relationship with the immune microenvironment and the regulation of target gene expression by non-coding RNAs in II/R injury. Therefore, we obtained datasets of II/R injury from public databases and screened for hub genes associated with immune infiltration and autophagy through biological analysis. Additionally, we constructed a nomogram model to predict the diagnosis of II/R injury and developed a circRNA–miRNA-mRNA regulatory network for these hub genes. We also assessed the therapeutic potential of the hub genes through drug prediction. Finally, qRT-PCR validation confirmed the relevance of these hub genes in II/R injury, providing new insights into the pathogenesis of this condition.

## 2 Materials and methods

### 2.1 Data source and processing

**FIGURE 1:**
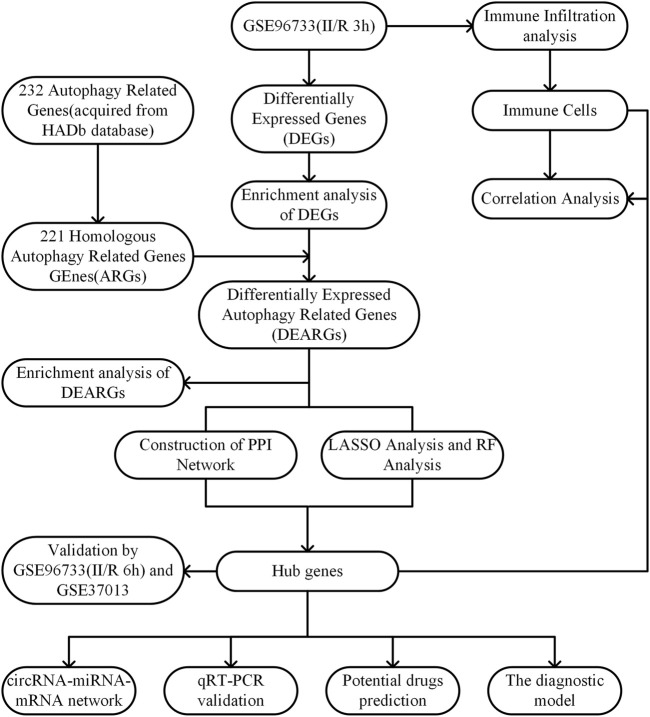
Experimental design roadmap.

The raw data of GSE96733 [species: *Mus musculus*; Platforms: GPL23038 (Clariom_S_Mouse) Affymetrix Clariom S Assay, Mouse (Includes Pico Assay)] was collected from the GEO website (http://www.ncbi.nlm.nih.gov/geo/). The GSE96733 (II/R 3 h) comprised four sham-operated samples and four II/R samples collected at 3 h (n = 4). The R software package “limma” was utilized to analyze the differentially expressed genes (DEGs) between sham-operated samples and those with II/R injury, with thresholds set at |log2FC| ≥ 1 and an adjusted *P*-value <0.05 for screening. Additionally, four sham-operated samples and four II/R samples at 6 h (n = 4) were included in GSE96733 (II/R 6 h) as a validation set. The human intestinal II/R injury expression profile microarray GSE37013 was also obtained from the GEO database to serve as a second validation set. From this dataset, we selected 21 samples, which included 7 human jejunum samples (sham), 7 human jejunum-reperfusion 30 min samples (II/R 30 min), and 7 human jejunum-reperfusion 120 min samples (II/R 120 min), and the platform used was GPL6947 Illumina HumanHT-12 V3.0 expression beadchip. The workflow of this research is shown in Figure 1.

### 2.2 Functional enrichment analysis of DEGs

The Gene Ontology (GO) analysis and Kyoto Encyclopedia of Genes and Genomes (KEGG) pathway enrichment of DEGs were performed using the R software (version 4.2.2) package “clusterProfiler” (Wu et al., 2021). The GO annotation encompasses three components: molecular function (MF), biological process (BP), and cellular component (CC). GO terms and signaling pathways exhibiting significant differences were identified under the criterion of adjusted *P*-value <0.05. Subsequently, the results were visualized using the R software packages “ggplot2″ and “GOplot” (Walter et al., 2015), respectively.

### 2.3 Identification and functional enrichment of differentially expressed autophagy-related genes

We identified 232 genes associated with autophagy from the Human Autophagy Database (http://www.autophagy.lu/). Since our study focuses on mice, we utilized the R software package “homologene” through the online platform Xiantao Academic (https://www.xiantaozi.com/) to conduct homologous gene conversion and obtain mouse homologous autophagy-related genes (ARGs). Subsequently, we intersected the ARGs with DEGs to identify differentially expressed autophagy-related genes (DEARGs). The differential expression of DEARGs was visualized using the R software package “ggpubr”. The R software package “Corrplot” was utilized to examine the Spearman correlation among DEARGs. KEGG and GO analyses of DEARGs were conducted using the R software package “clusterProfiler” with a significance threshold of *P* < 0.05.

### 2.4 PPI network analysis of DEARGs

The STRING online database (https://cn.string-db.org/) was employed to perform protein–protein interaction (PPI) network analysis, investigating the relationships among DEARGs with an interaction score exceeding 0.4. The resulting network was then analyzed and visualized using Cytoscape (version 3.10.3). To identify hub genes associated with autophagy, CytoHubba was employed, which calculated hub genes based on three distinct algorithms: Degree, Closeness, and Betweenness. Degree quantifies the number of direct connections a gene node has with other nodes, and genes with high degree values are typically positioned at the core of the network, possessing extensive biological functions or regulatory roles (Eisele et al., 2021). Closeness measures a gene’s ability to integrate information across the entire network by calculating the average shortest path distance from the node to all other nodes in the network. Genes with high closeness are generally located at the core of the network and can rapidly respond to environmental changes or regulate other genes (Park and Kim, 2009). Betweenness measures the frequency at which a node appears on the shortest paths connecting other nodes. Genes with high betweenness centrality are usually situated at the hubs of biological networks and may be involved in core regulatory or signaling processes (Roy et al., 2013). Therefore, we selected the top five genes from each algorithm as autophagy-related hub genes. Finally, the differential expression of these hub genes was analyzed using the Xiantao Academic online software.

### 2.5 Machine learning and identifying hub genes

To identify the hub genes and establish a diagnostic model of II/R injury, the least absolute shrinkage and selection operator (Lasso) algorithm, a logistic regression method for filtering variables to enhance the predictive performance, was initially adopted in this work to screen the hub genes with the “glmnet” package (Friedman et al., 2010). Next, the random forest (RF) algorithm, integrating multiple trees through the idea of ensemble learning to gain better accuracy, was employed to narrow down the hub genes with the “randomForest” package as well (Liaw and Wiener, 2002). We selected genes with |standardized regression coefficient| ≥ 0.05 in Lasso and feture importance score ≥0.1 in RF. The hub genes were subsequently determined by taking the union of these selected genes and those identified through PPI screening.

### 2.6 Construction and verification of the diagnostic model

The nomogram was constructed based on the five hub genes using the “rms” package (Zhao and Li, 2023). The area under the receiver operating characteristic (ROC) curve was plotted with the “pROC” R package to evaluate the performance of the hub genes in diagnosing II/R injury (Hanley and McNeil, 1982). Subsequently, the expression of the five hub genes was validated using the GSE37013 validation dataset, and the accuracy of these hub genes was assessed using the ROC curve.

### 2.7 Validation of hub genes expression in other datasets

The expression levels of key II/R injury genes were extracted from the GSE96733 (II/R 6 h) and GSE37013 datasets and analyzed using the t-test. The expression of hub genes was assessed, and the results are presented as violin plots. The results were visualized using the R software package “ggplot2”.

### 2.8 Immune cell in filtration analysis

The CIBERSORT algorithm is employed to evaluate changes in immune cells during immune infiltration. This study applies the algorithm to investigate alterations in the relative proportions of immune cell infiltration in II/R injury. Correlation analysis is conducted on 25 types of infiltrating immune cells in mice, and the results are visualized using the “ggcorrplot” package (Chen et al., 2017).

### 2.9 Construction of the circRNA-miRNA-mRNA regulatory network

This study initially analyzed five hub genes using the TargetScan (https://www.targetscan.org/vert_80/) and miRDB (https://mirdb.org/) databases. Subsequently, we employed RNAhybrid software (https://bibiserv.cebitec.uni-bielefeld.de/rnahybrid) to predict the circRNAs targeted by these miRNAs. Finally, the interaction network among the aforementioned genes, miRNAs, and circRNAs was visualized and plotted using Cytoscape software.

### 2.10 Predicting potential drugs

Utilize the Comparative Toxicogenomics Database (CTD) (https://ctdbase.org/) to predict targeted drugs for five key genes. Subsequently, visualize the results using the “ggplot2” package in R software.

### 2.11 II/R injury model construction

Before constructing the mouse II/R injury model, all mice were fasted for 12 h with free access to water. The mice were anesthetized via intraperitoneal injection of pentobarbital at a dosage of 30 mg/kg. Upon exhibiting signs of limb weakness, the mice were placed flat on a sterile drape, secured, and the abdominal skin was prepared and disinfected. Following local infiltration with 2% lidocaine, a 1.5 cm incision was made along the midline of the abdomen, approximately 2 mm below the xiphoid process, using a scalpel. The abdominal cavity was then accessed layer by layer to expose the intestines, and the superior mesenteric artery (SMA) was identified and isolated. A non-traumatic microarterial clamp was employed to temporarily occlude the SMA and its branches, with timing initiated immediately. The cessation of arterial pulsation was observed, and the intestinal tract became edematous and pale, indicating successful ischemia. The intestine was gently returned to the abdominal cavity, covered with sterile gauze, and the intestinal lumen was kept moist. After 45 min, the microarterial clamp was removed to restore blood flow. The return of the intestine’s color to a healthy red and the gradual strengthening of arterial pulsations indicated successful reperfusion. Subsequently, 0.5 mL of warm saline was dripped into the abdominal cavity, and the mouse’s abdomen was sutured layer by layer using 3–0 silk thread. The mouse was then placed in a cage adjacent to a radiator for warmth. Following 3 h of perfusion, all mice were euthanized with an overdose of pentobarbital (intraperitoneal injection, 200 mg/kg). The small intestine tissue was subsequently excised and stored at −80°C. For the sham surgery group, only the SMA was separated without clamping, and the same procedures as described above were followed (Gubernatorova et al., 2016).

### 2.12 Quantitative reverse transcription-polymerase chain reaction

Total RNA was extracted from frozen intestinal tissue using an RNA extraction kit (Thermo Fisher, USA, product number 15596-018). Subsequently, reverse transcription was performed using a cDNA synthesis kit (TIANGEN, China, product number SB-Q204) (Watson et al., 2008). The relative mRNA expression levels were evaluated using the 2^−ΔΔCq^ method, with β-actin serving as the internal control on the LightCycle 96 real-time fluorescence quantitative PCR system (Roche). Table 1 presents the primer sequences utilized.

**TABLE 1:** Primer sequences used in the qRT-PCR experiments.

Gene	Forward primer (5′–3′)	Reverse primer (3′–5′)
β-actin	TATGGAATCCTGTGGCATC	GTGTTGGCATAGAGGTCTT
Sqstm1	ATTGAGGTTGACATTGAT	TGTTACTCTTGTCTTCTG
Myc	ATGATGACCGAGTTACTT	GATGATGATGTTCTTGATGA
Hif1a	GCGGCGAGAACGAGAAGAAA	GGGGAAGTGGCAACTGATGA
Gabarapl1	CCAGTTGTGGCAGGAGACAT	GCAATCATAACCGTTCCCGC
Zfyve1	AGGAAGAAGATGACAGAGA	CAGGCTTACAGTCCAATT

### 2.13 Statistical analysis

Statistical analyses were performed using R software (version 4.4.2) and GraphPad Prism (version 9.0.0.121). The comparison of gene expression in II/R injury between the two groups was conducted using an unpaired Student's t-test. A *P*-value of less than 0.05 was deemed statistically significant in all analyses.

## 3 Results

### 3.1 DEGs identification

**FIGURE 2:**
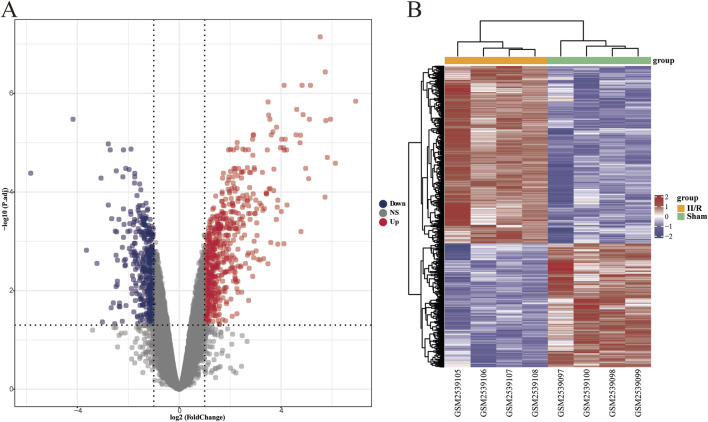
Identification of DEGs. **(A)** The volcano plot illustrates the differentially expressed genes between the Sham and II/R injury samples. Red dots represent upregulated genes, while dark blue dots indicate downregulated genes, with thresholds set at |log2FoldChange| ≥ 1 and an adjusted *P*-value <0.05. **(B)** The heatmap displays the differentially expressed genes between the Sham and III/R injury samples. Red signifies significantly upregulated DEGs, whereas dark blue indicates significantly downregulated DEGs in the samples.

Using the R software package “limma” for differential gene analysis, we identified a total of 1,027 DEGs from sham-operated samples and samples with II/R injury, comprising 605 upregulated genes and 422 downregulated genes (Figures 2A,B). See Supplementary Table S1 for details.

### 3.2 Enrichment analysis of DEGs

**FIGURE 3:**
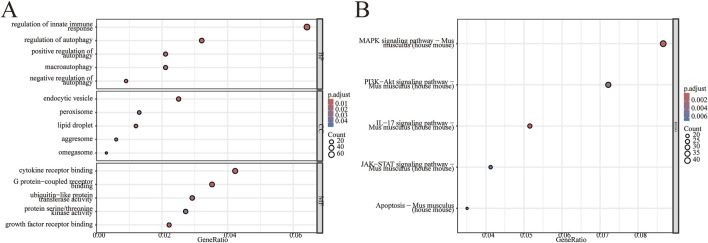
Enrichment analysis of DEGs. GO enrichment analysis was conducted on 1,027 differentially expressed genes, encompassing biological processes (BP), cellular components (CC), and molecular functions (MF). **(A)** The bubble plot illustrates the enriched GO terms. **(B)** The bubble plot displays the enriched KEGG pathways. GO, Gene Ontology; KEGG, Kyoto Encyclopedia of Genes and Genomes.

To further investigate the potential biological changes associated with the screened DEGs, GO and KEGG pathway enrichment analyses were conducted using R software. The results from the GO biological process (BP) annotation indicated that the DEGs were predominantly enriched in immune response and autophagy (Figure 3A). Furthermore, the KEGG pathway enrichment analysis revealed that the DEGs were primarily enriched in the MAPK signaling pathway, PI3K−Akt signaling pathway, JAK−STAT signaling pathway, as well as pathways related to apoptosis and IL-17 signaling pathway (Figure 3B). These findings suggest a significant correlation between II/R injury and autophagy. See Supplementary Table S2 for details.

### 3.3 Identification of DEARGs

**FIGURE 4:**
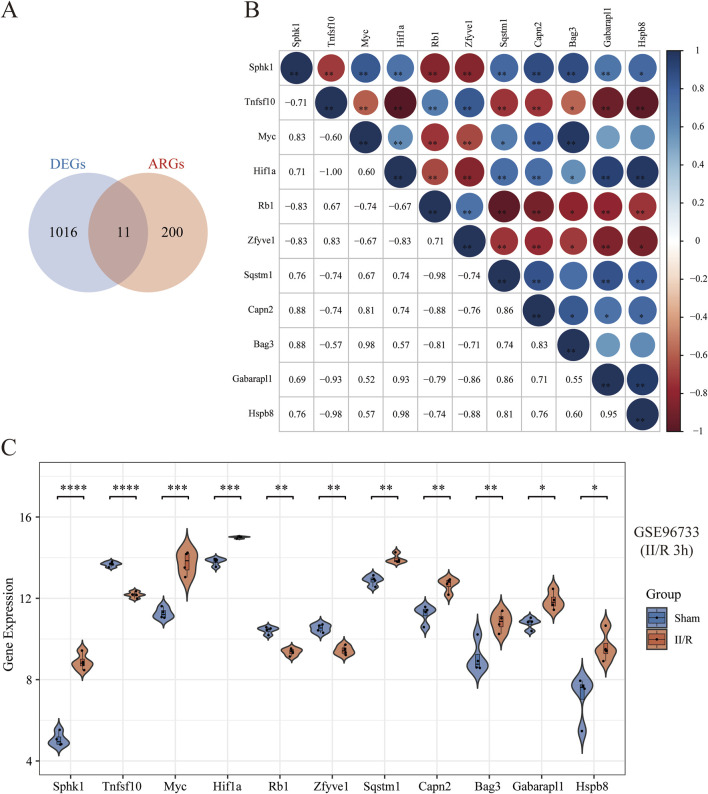
Identification of DEARGs. **(A)** Venn diagram of DEARGs between DEGs and ARGs. **(B)** Spearman’s correlation analysis of the 11 DEARGs. The abscissa and ordinate represent genes, different colors represent different correlation coefficients (blue represents positive correlation, and red represents negative correlation). The darker the color, the stronger the relation. Asterisks (*) stand for significance levels, *P < 0.05; **P < 0.01; ***P < 0.001. **(C)** Violin plot illustrate the differential expression of 11 autophagy-related genes in both II/R and Sham samples. The significance levels indicated as follows: *P < 0.05; **P < 0.01; ***P < 0.001; ****P < 0.0001. ns, not significant.

**FIGURE 5:**
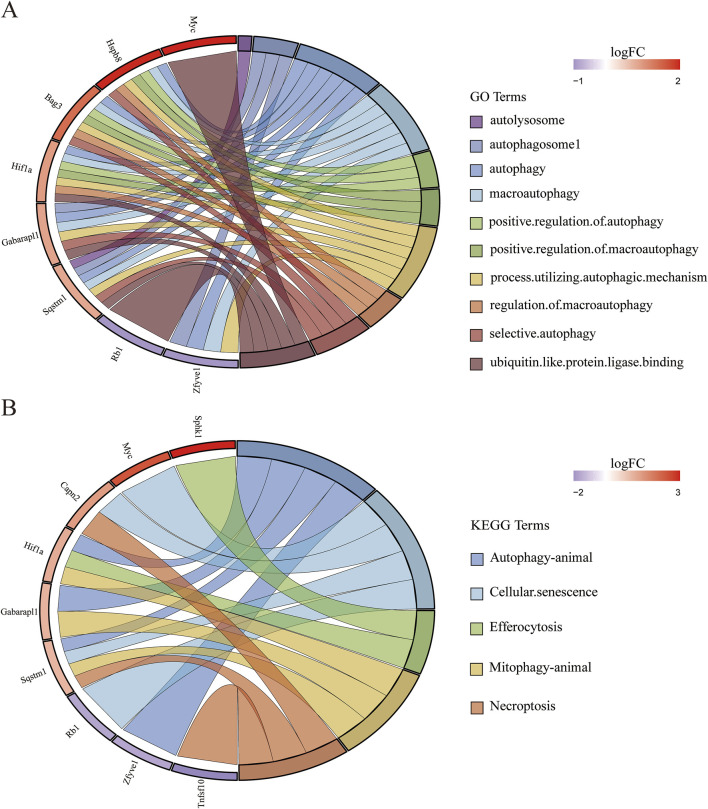
Gene-pathway association analysis in circos plots highlighting significant biological processes. **(A)** GO enrichment results of DEARGs. **(B)** KEGG enrichment results of DEARGs.

To further investigate autophagy-related genes in II/R injury, we retrieved 232 genes associated with autophagy from the Human Autophagy Database (http://www.autophagy.lu/). Given that our study focuses on mice, we utilized the R software package “homologene” on the Xiantao Academic online platform (https://www.xiantaozi.com/) to perform homologous gene conversion, thereby obtaining mouse homologous ARGs. By intersecting ARGs with DEGs, we identified 11 DEARGs (Figure 4A). A detailed correlation analysis was conducted on the expression levels of these 11 DEARGs within the dataset, revealing complex interactions among these genes (Figure 4B). Furthermore, utilizing the differential expression previously analyzed by the “limma” package, the R software package “ggpub” was employed to display the expression patterns of the 11 DEARGs in II/R injury compared to Sham samples (Figure 4C). The eight genes with increased expression were Sphk1, Myc, Hif1a, Sqstm1, Capn2, Bag3, Gabarapl1 and Hspb8, while the three genes with decreased expression were Tnfsf10, Rb1, Zfyve1 (Table 2). KEGG and GO analyses of the DEARGs were conducted with a significance threshold of *P* < 0.05, and the enrichment results were visualized using R software (Figures 5A,B). See Supplementary Table S3 for details.

**TABLE 2:** The 11 DEARGs in II/R injury samples compared to Sham samples.

Gene Symbol	logFC	T	P.Value	adj.P.Val	Changes
Sphk1	3.821317	16.62632425	3.72E-09	4.85E-06	Up
Myc	2.4811625	9.399595024	1.34E-06	0.000204443	Up
Hif1a	1.19521	8.430675209	3.89E-06	0.000399785	Up
Sqstm1	1.054035	6.130942141	7.33E-05	0.002664608	Up
Capn2	1.444815	5.999336797	8.85E-05	0.003039213	Up
Bag3	1.763602	4.905273791	0.000464499	0.009118529	Up
Gabarapl1	1.0979825	4.816769544	0.000535119	0.010086941	Up
Hspb8	2.454751	4.757286685	0.000588881	0.010733221	Up
Tnfsf10	−1.5074425	−10.63763978	3.89E-07	9.02E-05	Down
Rb1	−1.0709025	−7.010518618	2.21E-05	0.001239382	Down
Zfyve1	−1.083267	−6.594622326	3.85E-05	0.001807225	Down

### 3.4 Construction of PPI and identification of hub genes

**FIGURE 6:**
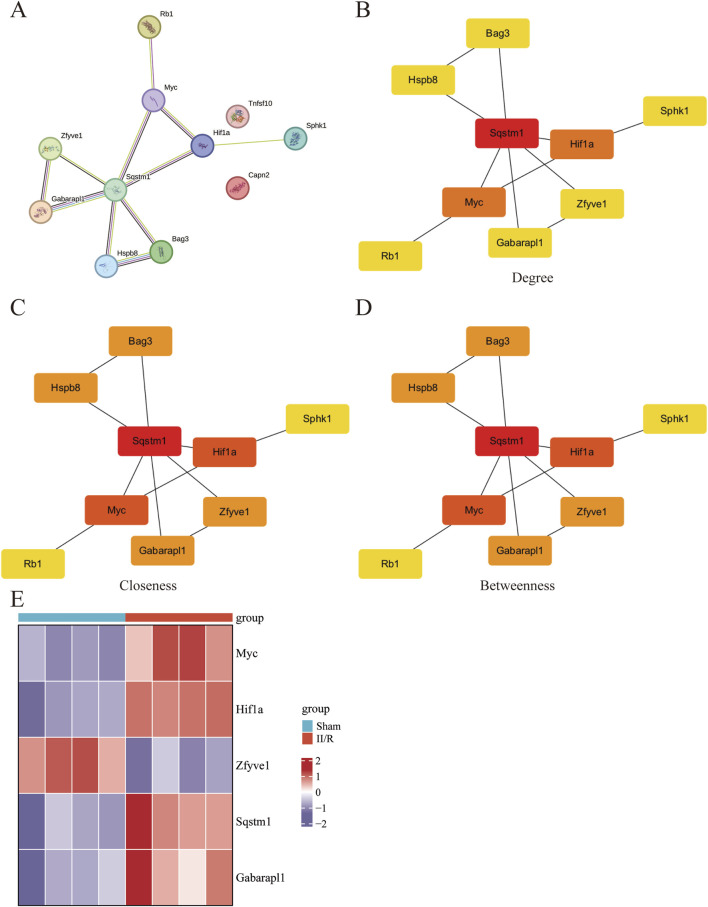
Screening of hub gene in DEARGs. **(A)** The protein–protein interaction network visualized by STRING. **(B–D)** The PPI network constructed from 11 DEARGs was analyzed using three algorithms: Degree, Closeness, and Betweenness. The top five genes were identified as hub genes. **(E)** The expression levels of Sqstm1, Myc, Hif1a, Gabarapl1, and Zfyve1 are illustrated using a heatmap.

We explored the interactions among 11 DEARGs using the medium-confidence STRING database, resulting in a PPI network comprising 11 nodes and 11 edges (Figure 6A). Subsequently, we calculated the PPI network of these 11 DEARGs using three algorithms (Degree, Closeness, and Betweenness) with the CytoHubba plugin, selecting the top five genes as hub genes (Figures 6B–D). These hub genes include Sqstm1, Myc, Hif1a, Gabarapl1, and Zfyve1, which are represented in a heatmap format (Figure 6E). See Supplementary Table S4 for details.

### 3.5 Machine learning screened hub genes and confirmed the final hub genes

**FIGURE 7:**
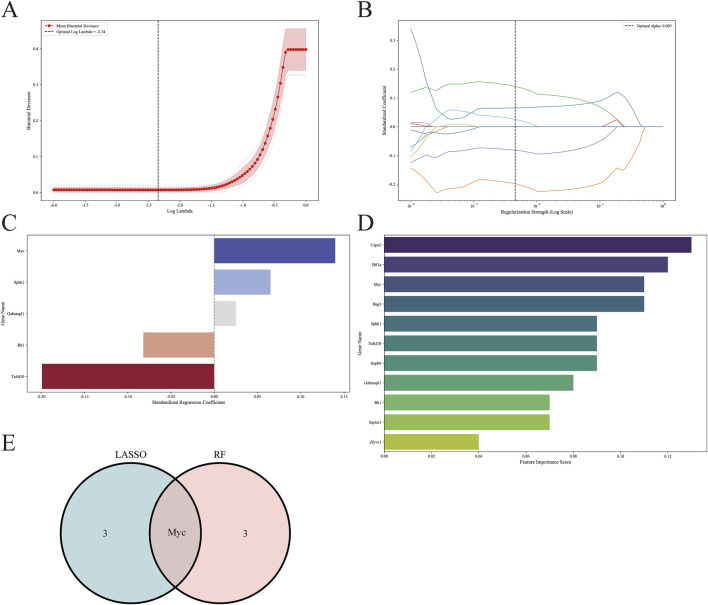
Screening for hub genes by machine learning algorithms. **(A–C)** Hub genes identified using the least absolute shrinkage and selection operator (Lasso) logistic regression algorithm. **(D)** Random forest (RF) algorithm to screen hub genes. **(E)** Venn diagram demonstrating overlapping hub gene screened by Lasso and RF.

We employed machine learning algorithms to screen hub genes from DEARGs, initially utilizing the Lasso algorithm to identify four candidate hub genes based on |standardized regression coefficient| ≥ 0.05. Subsequently (Figures 7A–C). we applied the RF algorithm to select four candidate hub genes with feature importance score ≥0.1 (Figure 7D). The common gene Myc was identified through the intersection of a Venn diagram (Figure 7E). Finally, we took the union of the genes selected based on the PPI network and determined the final hub genes to be Sqstm1, Myc, Hif1a, Gabarapl1, and Zfyve1.

### 3.6 Construction and verification of the diagnostic model

**FIGURE 8:**
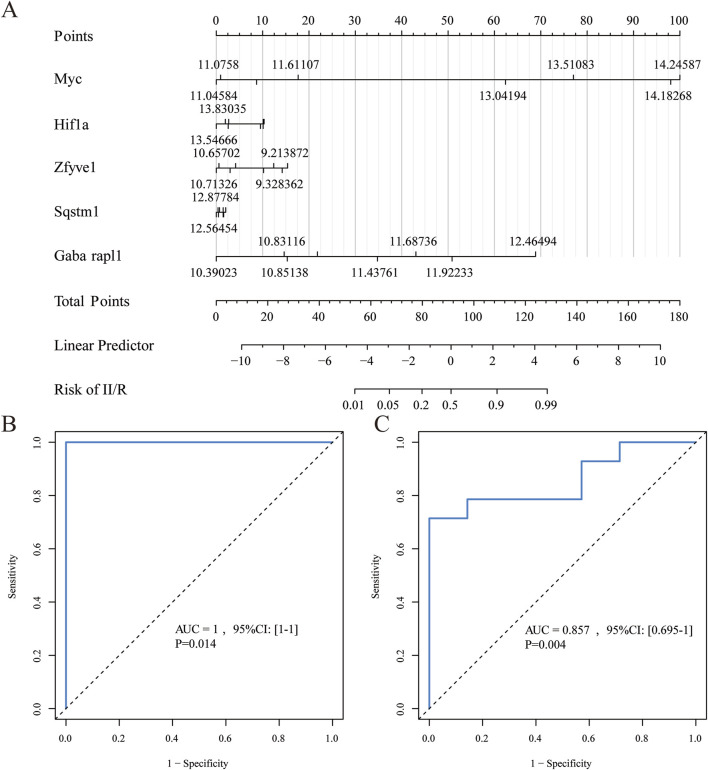
Diagnostic model construction. **(A)** Construction of a nomogram model with 5 hub genes. **(B,C)** ROC curve for evaluating and validating the diagnostic model’s performance.

We utilized five identified hub genes (Sqstm1, Myc, Hif1a, Gabarapl1, and Zfyve1) as diagnostic markers for predicting II/R injury and constructed a nomogram (Figure 8A). The diagnostic performance was evaluated using ROC curves, achieving an area under the curve (AUC) value of 1 in the training set (Figure 8B). The efficiency of the predictive model was further validated using the GSE37013 dataset, which yielded an AUC value of 0.857 (95% CI = 0.695-1) (Figure 8C), indicating that the diagnostic model possesses good predictive value for II/R injury. However, the accuracy and reliability of this diagnostic model still require further investigation in future clinical trials. See Supplementary Table S5 for details.

### 3.7 Validation of hub genes expression in other datasets

**FIGURE 9:**
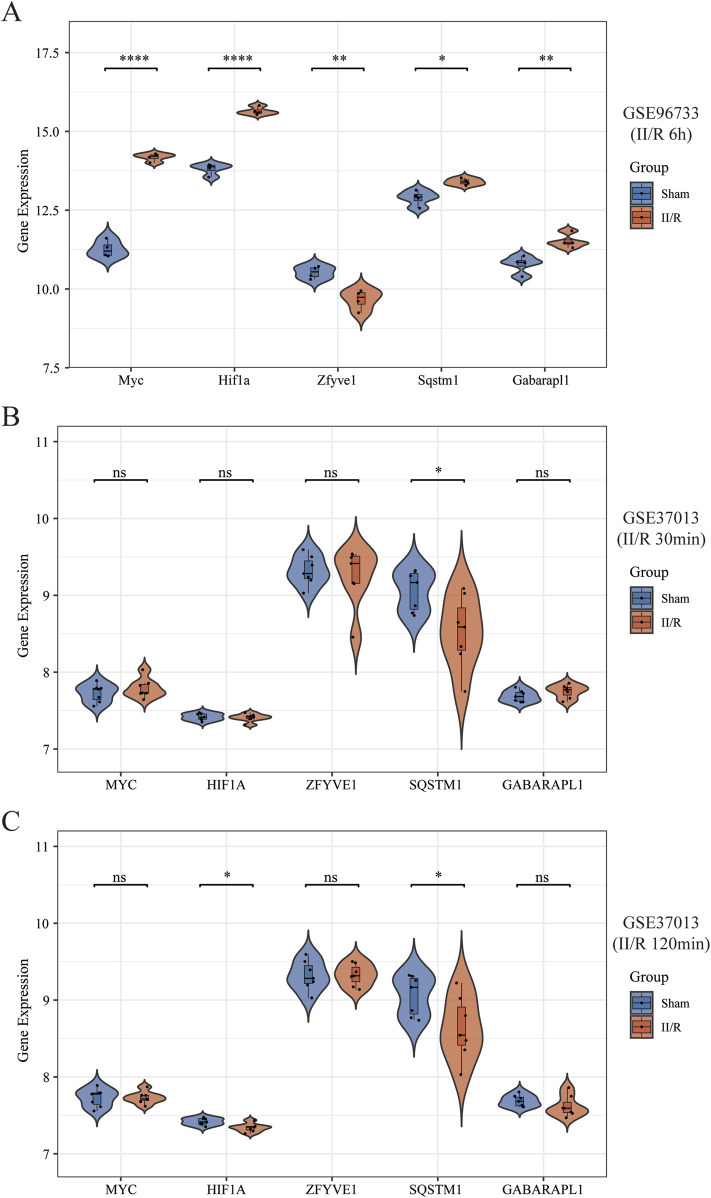
Validation of hub genes expression in other datasets. **(A–C)** The expression levels of Sqstm1, Myc, Hif1a, Gabarapl1, and Zfyve1 were verified by GSE96733 (II/R 6 h), GSE37013 (II/R 30 min), and GSE37013 (II/R 120 min) datasets, the results of which are presented as violin plot. The significance levels indicated as follows: *P < 0.05; **P < 0.01; ***P < 0.001; ****P < 0.0001. ns, not significant.

We selected the data from GSE96733 (II/R 6 h) for validation and found that the expression of the five hub genes was consistent with the bioinformatics analysis of GSE96733 (II/R 3 h) (Figure 9A). Furthermore, upon validating with the GSE37013 dataset on human II/R injury, it was observed that the expression of SQSTM1 in the II/R group was lower than that in the sham group at both 30 min and 120 min of II/R injury (Figures 9B,C), indicating that autophagy plays a protective role in the early stages of II/R injury. See Supplementary Table S6 for details.

### 3.8 Immune cell analysis

**FIGURE 10:**
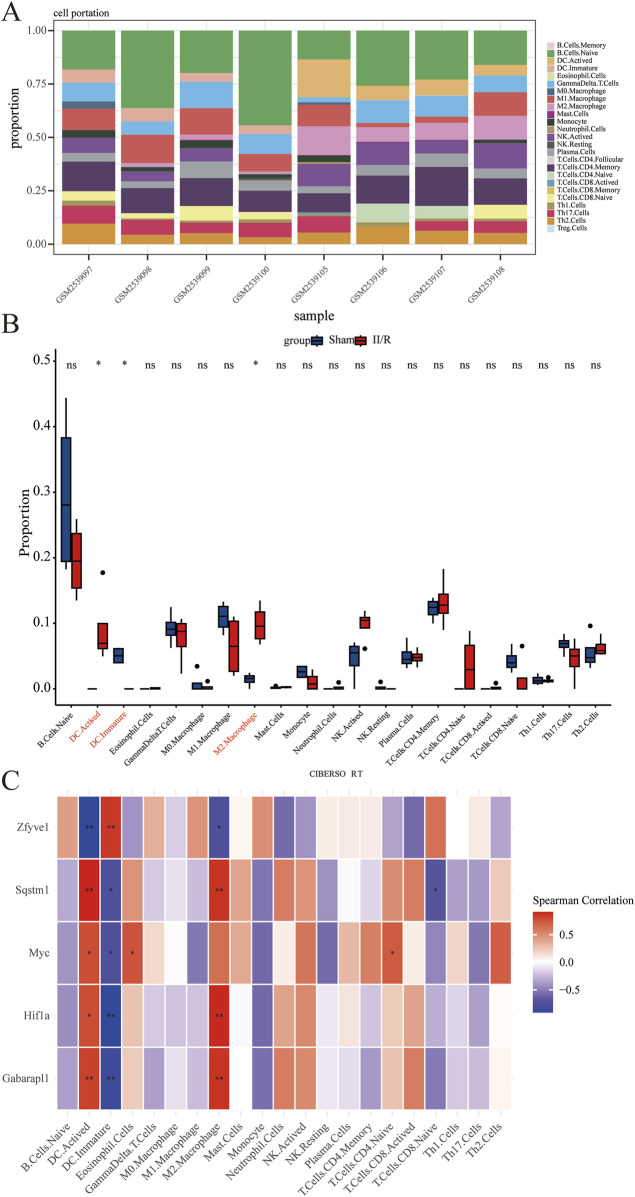
Results of immune cell analysis. **(A)** The histogram indicates the relative proportions of 25 immune cells. **(B)** Box plots illustrating the relative expression of each immune cell subtype between Sham and II/R injury samples. **(C)** Heatmap showing the correlation between immune cells and five hub genes. *P < 0.05; **P < 0.01.

We employed the CIBERSORT algorithm to assess the relative abundance of infiltrating immune cell subtypes in Sham samples and II/R injury samples. The bar graph illustrates the infiltration of various immune cell subtypes in each sample, while the box plot demonstrates the differences in the percentage of immune cells between the two sample groups. Compared to Sham, the II/R samples exhibited an increase in M2 macrophages (Figures 10A,B). Finally, the heatmap reveals the correlation between hub genes and immune cell infiltration, indicating that the expression of hub genes is significantly associated with immune cell infiltration among subtypes (Figure 10C). Notably, activated dendritic cells (DC.Actived) is negatively correlated with Zfyve1 and positively correlated with Sqstm1, Myc, Hif1a, and Gabarapl1. In contrast, the results for immature dendritic cells (DC.Immature) exhibit the opposite correlations. See Supplementary Table S7 for details.

### 3.9 Analysis of the circRNA-miRNA-mRNA regulatory network

**FIGURE 11:**
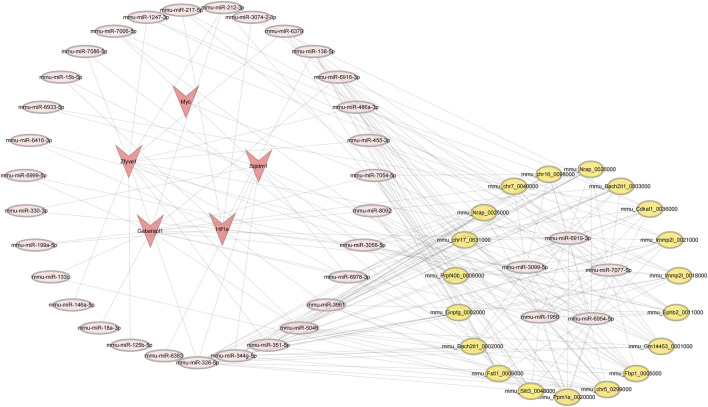
Results of the circRNA-miRNA-mRNA regulatory network. Red represents: mRNA; pink represents: miRNA; yellow represents: circRNA.

The analysis results indicate that several miRNAs (including mmu-miR-6954-5p, mmu-miR-6919-3p, mmu-miR-7077-5p, mmu-miR-3099-5p, and mmu-miR-1956) demonstrate significantly higher node connectivity within the regulatory network. Notably, the Myc proto-oncogene interacts significantly with only one miRNA (mmu-miR-7077-5p), and a total of 14 circRNAs that interact with mmu-miR-7077-5p have been identified (Figure 11). See Supplementary Table S8 for details.

### 3.10 Potential drugs prediction

**FIGURE 12:**
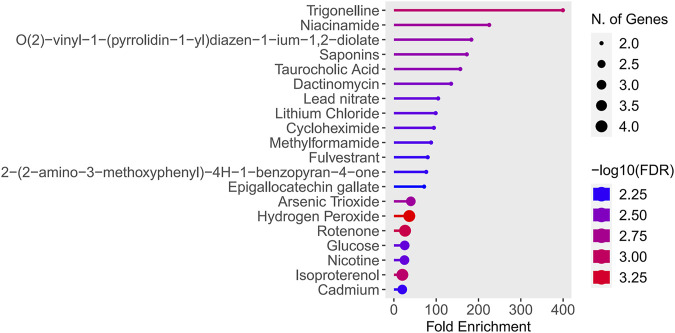
Results of drugs prediction.

The study found that Trigonelline and Niacinamide were particularly notable, as they exhibited both the highest enrichment factors and the lowest false discovery rates (FDR values) (Figure 12). See Supplementary Table S9 for details.

### 3.11 RT-qPCR verification of hub genes

**FIGURE 13:**
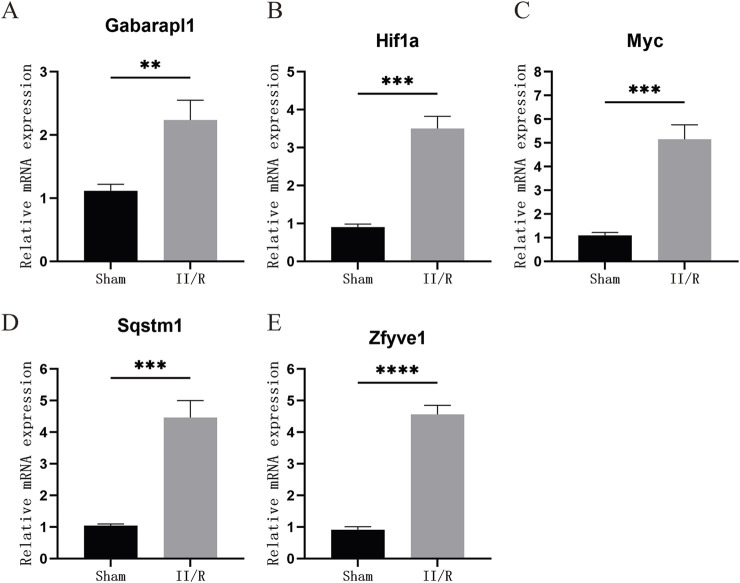
The expression levels of hub genes in the mouse II/R injury model. **(A–E)** The mRNA levels of Gabarapl1, Hif1a, Myc, Sqstm1, and Zfyve1 were assessed using qRT-PCR. *P < 0.05, **P < 0.01, ***P < 0.001, ****P < 0.0001. ns, not significant.

To validate the credibility of the GSE96733 dataset, we conducted qRT-PCR analysis on five autophagy-related genes. Compared to the Sham samples, the expression levels of Myc, Hif1a, Sqstm1, and Gabarapl1 were significantly increased in the II/R injury samples, aligning with the results from the bioinformatics analysis (Figure 13). See Supplementary Table S10 for details.

## 4 Discussion

II/R injury is a severe clinical condition, yet treatment options remain limited (Nadatani et al., 2018). Developing innovative therapeutic strategies is of significant importance in alleviating II/R injury. In previous studies, the autophagy marker Sqstm1/p62 was downregulated during a phase of 45 min of intestinal ischemia followed by 2 h of reperfusion. During this phase, the activation of autophagy plays a crucial protective role by clearing damaged mitochondria and misfolded proteins, with the mechanism involving the coordinated regulation of multiple molecular pathways (Chen et al., 2020). In the study of Corilagin’s effects on II/R injury, the degradation of Sqstm1 was significantly increased, while the expression of autophagy-related proteins (such as LC3II and Beclin1) was upregulated, suggesting that Corilagin activates autophagic flux through the AMPK/Sirt1 pathway, thereby alleviating oxidative stress and cell apoptosis to exert a protective effect (Li et al., 2023). At the stage of 45 min of intestinal ischemia followed by 4 h of reperfusion, autophagic flux is disrupted (e.g., the fusion of autophagosomes with lysosomes is blocked), leading to the accumulation of Sqstm1 within cells, which is manifested as an upregulation at the protein level. At this point, autophagy shifts from a protective mechanism to a damage-promoting factor (Wang et al., 2019; Wen et al., 2019). Therefore, we selected samples from GSE96733 with 45 min of intestinal ischemia followed by 3 h of reperfusion for our study, indicating that this period represents dynamic changes in autophagy. In the GSE37013 human dataset, whether subjected to 30 min or 120 min of intestinal ischemia-reperfusion, SQSTM1 levels in the II/R group were found to be lower than those in the sham group. This observation indirectly confirms the protective role of autophagy during this phase. It suggests that the 3-h mark may represent a critical turning point, where autophagy transitions from a protective mechanism to a pro-injury factor in II/R injury. However, the relationship between autophagy and II/R injury is not yet fully understood. Additionally, the application of bioinformatics analysis in the context of ARGs in II/R injury has not been thoroughly explored. Therefore, we conducted this study to investigate whether the expression of ARGs in II/R injury significantly differs from that in the sham group. Additionally, we explored the correlation between II/R injury and immune infiltration, which aids in understanding the potential immune mechanisms underlying II/R injury and in identifying potential molecular targets for its treatment.

In this study, we conducted KEGG and GO enrichment analyses using 1,027 DEGs obtained from the database. The KEGG results indicated a significant activation of immune-related disease pathways in II/R injury, particularly the IL-17 signaling pathway. Previous studies have suggested that regulating the production of IL-17 may be advantageous for mitigating tissue damage induced by II/R injury (Edgerton et al., 2009).

GO results indicated that autophagy plays a central role in the development of ischemia/reperfusion (II/R) injuryConsequently, based on these findings, we screened for autophagy-related genes among the DEGs. Subsequently, by utilizing the Lasso algorithm, RF algorithm, and PPI network, we ultimately identified five hub genes: Myc, Hif1a, Zfyve1, Sqstm1, and Gabarapl1. The expression levels of these five hub genes in the raw data showed significant differences between the sham-operated samples and the II/R injury samples. Furthermore, we validated these findings using GSE96733 (II/R 6 h), and the results were consistent with the original bioinformatics analysis. Additionally, we constructed a diagnostic model for II/R injury, which demonstrated good diagnostic efficacy when validated with GSE37013.

CIBERSORT is an inverse deconvolution analysis algorithm based on linear support vector regression that estimates the relative abundance of immune cells in mixed cell populations by analyzing gene expression data (Newman et al., 2015). Using this algorithm, we found that M2 macrophages and activated dendritic cells (DCs) were more abundant in II/R injury samples compared to sham-operated samples. Through correlation analysis of hub genes, we discovered that activated DCs were negatively correlated with Zfyve1 and positively correlated with Sqstm1, Myc, Hif1a, and Gabarapl1. In contrast, the results for immature DCs were opposite to those findings. For M2 macrophages, Zfyve1 was negatively correlated with them, while Sqstm1, Hif1a, and Gabarapl1 were positively correlated.

Macrophages, integral components of the innate immune system, exhibit significant plasticity, allowing them to adjust their phenotypes in response to environmental stimuli. Under homeostatic conditions, macrophages serve a protective role in maintaining the intestinal barrier (Ruder and Becker, 2020). Our earlier research indicated that promoting the transformation of M1 macrophages to M2 may mitigate II/R injury (Liu et al., 2015). Recent studies have demonstrated that enhancing the release of interleukin-10 by M2 macrophages through Toll-like receptor 2 signaling can alleviate II/R injury (Hu et al., 2022). It has been shown that Toll-like receptor (TLR) signaling activates the Nrf2 pathway and induces autophagy via p62, a downstream target of Nrf2. Furthermore, another study revealed that Nrf2 prevents acute lung injury induced by II/R injury by modulating TLR4 signaling and inducing autophagy (Yan et al., 2018). We can hypothesize that Nrf2 may ameliorate II/R injury by regulating TLR-induced autophagy and promoting M2 macrophages, a proposition that necessitates further research for validation. This provides a theoretical basis and new therapeutic strategies for the treatment of II/R injury. Dendritic cells (DCs) serve as crucial regulators of intestinal immunity. Previous studies have demonstrated that II/R injury increases the infiltration of DCs into the small intestine and is associated with the upregulation of TLR4 (Hagiwara et al., 2010). These findings suggest that TLR4 plays a pivotal role in regulating autophagy and immune cells during II/R injury.

Research on non-coding RNAs in II/R injury is becoming increasingly extensive. Studies have demonstrated that inhibiting miR-379-5p can enhance epithelial cell proliferation and improve barrier function following II/R injury (Jia et al., 2023). Suppressing miR-665-3p may represent a potential clinical approach for anti-inflammatory and anti-apoptotic treatment of II/R injury (Li et al., 2018). Additionally, miR-26b-5p may prevent II/R injury by targeting DAPK1 and inhibiting apoptosis in intestinal mucosal cells (Zhou et al., 2022). Our study revealed that multiple microRNAs (including mmu-miR-6954-5p, mmu-miR-6919-3p, mmu-miR-7077-5p, mmu-miR-3099-5p, and mmu-miR-1956) exhibit significantly higher node connectivity within the regulatory network. The Myc oncogene shows a significant interaction with only a single miRNA (mmu-miR-7077-5p). This specific regulatory relationship suggests that selecting Myc as a subsequent validation target offers a unique advantage: it is less influenced by the miRNA regulatory network, thereby facilitating the establishment of a clear molecular regulatory mechanism research system. Currently, no one has studied this RNA, providing a new perspective for research on II/R injury.

Trigonelline is a naturally occurring polar hydrophilic alkaloid found in various plants, including raw coffee beans and fenugreek seeds. It exhibits multiple effects, such as anti-diabetic, neuroprotective, and anti-cancer properties, and it modulates oxidative stress by inhibiting the detrimental Nrf2 pathway when autophagy is compromised (Nguyen et al., 2024). Furthermore, studies have demonstrated that trigonelline inhibits autophagy via the PI3K/AKT/mTOR signaling pathway, thereby mitigating cerebral ischemia-reperfusion pyroptosis (Qiu et al., 2025). Although there is currently no research on the effects of trigonelline on II/R injury, this opens new avenues for drug treatment strategies. The interaction between niacinamide and autophagy has increasingly become a focus of recent research. Oral administration of nicotinamide mononucleotide (NMN) combined with aerobic exercise can elevate the levels of nicotinamide adenine dinucleotide (NAD) in mice and enhance mitophagy (Wu et al., 2023). This also provides theoretical support for studying II/R injury. Further exploration of the specific effects of trigonelline and niacinamide on gene expression and their mechanisms of action is essential, as this will provide a scientific foundation for developing treatments for II/R injury.

Finally, we conducted *in vitro* experiments to verify whether the hub genes were differentially expressed in the II/R injury mouse model. Based on the results from reverse transcription RT-qPCR, we found that the four hub genes—Sqstm1, Myc, Hif1a, and Gabarapl1—were differentially expressed in the II/R injury group compared to the sham group, with expression trends consistent with the previous bioinformatics analysis results.

Sqstm1/p62 (sequestosome 1) is a classical autophagy receptor that plays a crucial role in selective autophagy, facilitating the elimination of abnormal intracellular components and the recycling of bioenergy substrates (Huang et al., 2024). The elevated RT-qPCR results indicate that autophagic flux is disrupted at 3 h post-reperfusion in II/R injury, transforming autophagy into a pro-damage factor. This finding provides a solid theoretical basis for the early treatment of II/R injury. Myc, a key member of the Myc proto-oncogene family, regulates nearly all physiological processes in cells, including the cell cycle, proliferation, metabolism, differentiation, and apoptosis (Jha et al., 2023). II/R injury can induce distal lung tissue damage by activating oxidative stress and inflammatory responses, while melatonin may exert a protective effect against such damage by upregulating the expression of N-myc downstream regulated gene 2 (NDRG2) (Yang et al., 2015). Although the protective role of Myc in II/R injury has not been directly investigated, this offers indirect theoretical support for its involvement. Studies have demonstrated that hypoxia and hypoxia-inducible factors (Hifs) 1 and 2 alpha are involved in tumor immune escape (Wu et al., 2022). However, the role of Hif1a in the context of II/R injury has not been directly investigated. Chronic hypoxia-induced Hif1a enhances myocardial ischemia tolerance by promoting mitophagy (Alanova et al., 2024). II/R injury is often accompanied by mitochondrial dysfunction and cell death, and Hif1a may help maintain intestinal epithelial cell homeostasis by activating the autophagy pathway. Gabarapl1 (Gamma-aminobutyric acid receptor-associated protein-like 1) is a crucial member of the autophagy-related ATG8 protein family, playing a significant role in various physiological and pathological processes through the regulation of autophagy (Poillet-Perez et al., 2017). Research on Gabarapl1 in the context of II/R injury is limited; however, it may influence the survival of intestinal epithelial cells by regulating the integrity of the autophagic flux, which warrants further investigation.

This study inevitably has several limitations. Firstly, the sample size of the gene expression profiles obtained from public databases is somewhat insufficient, and the individual differences among the samples may influence the generalizability of the analysis results. Furthermore, RT-qPCR only validated the mRNA levels of the hub genes, without confirming the protein levels of these genes. More relevant *in vivo* and *in vitro* experiments are necessary to elucidate the roles of these hub genes and their potential mechanisms in II/R injury.

## 5 Conclusion

In summary, we employed bioinformatics methods to analyze the relationship between autophagy-related genes and II/R injury. This analysis led to the identification of four hub genes—Sqstm1, Myc, Hif1a, and Gabarapl1—that may serve as potential biomarkers for the diagnosis, mechanistic research, and treatment of II/R injury. Furthermore, we conducted an immune cell infiltration analysis, constructed a circRNA-miRNA-mRNA network for the hub genes, and performed potential drug predictions. These findings provide promising indications for identifying targets for subsequent research and treatment.

## Data Availability

The datasets presented in this study can be found in online repositories. The names of the repository/repositories and accession number(s) can be found in the article/Supplementary Material.
