# Supplementary material for: Identification of autophagy-related genes in intestinal ischemia-reperfusion injury and their role in immune infiltration
Source: Front Physiol. 2025 May 26;16:1601968. doi: 10.3389/fphys.2025.1601968 (PMC12146356; doi:10.3389/fphys.2025.1601968)
Supplement: Supplementary file 2 [file Table11.doc]

**Differentially expressed genes**

geo<-getGEO('GSE96733',destdir =".",getGPL = F)

anno=fread("GPL23038-70510.txt")%>%data.frame()

anno=anno[anno[,"mrna_assignment"]!="---",]%>%na.omit()

ID=unlist(str_split(anno$mrna_assignment,"[ //]",simplify=T))[,1]

anno=data.frame(anno$ID,ID)

id1=bitr(anno$ID,fromType = "REFSEQ",toType = c("SYMBOL"),OrgDb = org.Mm.eg.db)

id2=bitr(anno$ID,fromType = "ENSEMBLTRANS",toType = c("SYMBOL"),OrgDb = org.Mm.eg.db)

colnames(id1)=c("ID","SYMBOL")

colnames(id2)=c("ID","SYMBOL")

id=rbind(id1,id2)

probe2symbol=merge(anno,id,by="ID")%>%.[,-1]

colnames(probe2symbol)=c("ID","SYMBOL")

expr=data.frame(exprs(geo[[1]]))

expr$ID=rownames(expr)

data=merge(probe2symbol,expr,by="ID")

expr_mean=aggregate(.~SYMBOL,mean,data=data[,-1])

rownames(expr_mean)=expr_mean[,1]

expr_mean=expr_mean[,-1]

write.csv(expr_mean,"GSE96733.csv")

data=expr_mean[,c(1:4,9:12)]

list <- c(rep("sham", 4), rep("IR",4)) %>% factor(., levels = c("sham", "IR"), ordered = F)

list <- model.matrix(~factor(list)+0)

colnames(list) <- c("sham", "IR")

df.fit <- lmFit(data, list)

df.matrix <- makeContrasts(IR - sham, levels = list)

fit <- contrasts.fit(df.fit, df.matrix)

fit <- eBayes(fit)

tempOutput <- topTable(fit,n = Inf, adjust = "fdr")

n=1

tempOutput [which(tempOutput $logFC >= n & tempOutput $adj.P.Val< 0.05),'sig'] <- 'Up'

tempOutput [which(tempOutput $logFC <= -n & tempOutput $adj.P.Val < 0.05),'sig'] <- 'Down'

tempOutput [which(abs(tempOutput $logFC) <= n | tempOutput $adj.P.Val >= 0.05),'sig'] <- 'NoSignifi'

All_diffSig <- data.frame(subset(tempOutput , sig %in% c('Up', 'Down')))

write.csv(tempOutput, file = "All gene.csv")

write.csv(All_diffSig, file = "DEG.csv")

**Volcano plot (Figure 2A)**

tempOutput$SYMBOL <- rownames(tempOutput)

ggplot(tempOutput,aes(x=logFC,y=-log10(adj.P.Val),color=sig))+

geom_point(size=5,alpha=0.5)+

scale_color_manual(values=c("#00008B","#808080","#DC143C"))+

theme_bw()+

theme(

legend.title = element_blank(),

legend.text = element_text(size = 12),

axis.text.x=element_text(size=14,face="plain"),

axis.text.y=element_text(size=14,face="plain"),

axis.title.y=element_text(size = 14,face="plain"),

axis.title.x=element_text(size = 14,face="plain")

)+

ylab('-log10 (P.adj)')+

xlab('log2 (FoldChange)')+

geom_vline(xintercept=c(-1,1),lty=3,col="black",lwd=0.5) +

geom_hline(yintercept = -log10(0.05),lty=3,col="black",lwd=0.5)+

guides(color=guide_legend(override.aes = list(size=8,alpha=1)))

**Heatmap (Figure 2B)**

#data <- read.csv("热图1.csv",row.names = 1)

#group <- read.table("group.txt",sep = "\t",header = T,

row.names = 1)

#anno_colors <- list(group=c(Sham="#71c286",`II/R`="#fca718"))

#pdf("heatmap.pdf",width = 6,height = 8)

pushViewport(viewport(gp = gpar(fontfamily = "serif")))

ht =pheatmap(as.matrix(data),

annotation_col = group,

color = colorRampPalette(c("#414b81","#7167a1","white","#ac5648","#ce222d"))(100),

show_rownames = F,

annotation_colors = anno_colors,

fontfamily = "serif",

name= " ",

fontsize = 12,

scale = "row")

draw(ht, newpage = FALSE)

popViewport()

dev.off()

**The bubble plots of GO terms and KEGG pathways. (Figures 3A,B)**

# file_path <- "准备文件.txt"

# table_data <- read.table(file_path, sep = "\t", header = TRUE, check.names = FALSE)

# gene_column <- table_data[[1]]

# entrez_ids <- c()

# for (gene in gene_column) {

# id <- tryCatch({

# mget(gene, org.Mm.egSYMBOL2EG)

}, error = function(e) {

# NA

})

# entrez_ids <- c(entrez_ids, id)

}

# entrez_ids <- as.character(entrez_ids)

# table_data <- cbind(table_data, entrezID = entrez_ids)

# table_data <- table_data[!is.na(table_data[, "entrezID"]), ]

# gene <- table_data$entrezID

# GO <- enrichGO(gene = gene,

OrgDb = org.Mm.eg.db,

pvalueCutoff = 0.05,#

qvalueCutoff = 0.05,#

ont = "all",

readable = TRUE)

# write.table(GO, file = "GO.txt", sep = "\t", quote = FALSE, row.names = FALSE)

####

interest_terms <- c("GO:0045088", "GO:0010506", "GO:0010508", "GO:0010507",

"GO:0016236", "GO:0030139", "GO:0005811", "GO:0005777",

"GO:0016235", "GO:1990462", "GO:0005126", "GO:0001664",

"GO:0019787", "GO:0070851", "GO:0004674")

# selected_GO <- subset(GO, GO$ID %in% interest_terms)

new_GO <- GO

# new_GO@result <- selected_GO

### pdf("GO-气泡图.pdf", width = 13, height = 10)

# if (length(new_GO) > 0) {

# dotplot(new_GO, showCategory = 5, split = "ONTOLOGY", orderBy = "GeneRatio") +

# facet_grid(ONTOLOGY ~ ., scale = "free")

} else {

# cat("\n")

}

# dev.off()

# KEGG <- enrichKEGG(gene = gene,

organism = "mmu",

pvalueCutoff = 1,

qvalueCutoff = 1)

# KEGGG <- as.data.frame(KEGG)

KEGGG <- KEGGG[, !names(KEGGG) %in% "category"]

# names(KEGGG)[names(KEGGG) == "subcategory"] <- "ONTOLOGY"

# KEGGG$ONTOLOGY <- "KEGG"

KEGG2 <- KEGG

KEGG2@result <- KEGGG

write.table(KEGG, file = "KEGG.txt", sep = "\t", quote = FALSE, row.names = FALSE)

interest_terms2 <- c("mmu04010", "mmu04151", "mmu04630",

'mmu04657','mmu04210')

# selected_GO2 <- subset(KEGG2, KEGG2$ID %in% interest_terms2)

# KEGG2@result <- selected_GO2

### pdf("KEGG-气泡图.pdf", width = 13, height = 10)

# if (length(KEGG2) > 0) {

# dotplot(KEGG2, showCategory = 5, split = "ONTOLOGY", orderBy = "GeneRatio") +

#

facet_grid(ONTOLOGY ~ ., scale = "free")

} else {

#

cat("\n")

}

# dev.off()

**Spearman's correlation analysis (figure 4B)**

#data <- read.csv("箱式图2 (1).csv",header = T,row.names = 1)

data <- as.data.frame(t(data))

#res <- cor(data,

method = "spearman")

res1 <- cor.mtest(data) #

#pdf("cor.pdf",width = 10,height = 8)

corrplot(res, type = 'upper', tl.pos = 'tp',method = "circle",

tl.col = "black",

p.mat = res1$p, #

insig = "label_sig",

sig.level = c(0.01,0.05),

pch.cex = 2) #

corrplot(res, add = TRUE, type = 'lower', method = 'number',

diag = FALSE,

tl.pos = 'n',

cl.pos = 'n',

col = "black") #

dev.off()

**The circos plots of GO terms and KEGG pathways. (Figures 5A,B)**

DEG <- read.table("准备文件 (1).txt",sep = "\t",check.names = F,stringsAsFactors = F,header = T)

colnames(DEG)[1] <- "Gene"

gene = DEG$Gene

GO_database <- 'org.Mm.eg.db' #

KEGG_database <- 'mmu' #

http://www.genome.jp/kegg/catalog/org_list.html

trans <- bitr(gene, fromType="SYMBOL",

toType="ENTREZID", OrgDb="org.Mm.eg.db")

#######################GO

ego <- enrichGO(gene = trans$ENTREZID,

OrgDb = org.Mm.eg.db,

ont = "ALL",

pAdjustMethod = "BH",

pvalueCutoff = 0.5,

qvalueCutoff = 1,

readable = TRUE)

head(ego@result)

result<-ego@result

write.table(result, file='GO result.txt',sep = "\t",row.names = T,col.names = NA,quote = F)#

#organism supported organism listed in 'http://www.genome.jp/kegg/catalog/org_list.html'

#hsa ；mmu

kk <- enrichKEGG(gene = trans$ENTREZID,

organism = 'mmu',

pvalueCutoff = 0.5,

qvalueCutoff = 1)

tmp <- data.frame(kk)

kkx <- setReadable(kk, 'org.Mm.eg.db', 'ENTREZID')

kk<- data.frame(kkx)

write.table(kk, file='kegg.result1.txt',sep = "\t",row.names = T,col.names = NA,quote = F)#

# get top 6 terms for visualization

ego1_df <- data.frame(ego) %>%

group_by(ONTOLOGY) %>%

arrange(pvalue) %>%

slice_head(n = 5) %>%

rowwise() %>%

mutate(fc = eval(parse(text = GeneRatio))/eval(parse(text = BgRatio)))

# plot

library(circlize)

# colors for ONTOLOGY group

col <- rep(rand_color(n = length(unique(ego1_df$ONTOLOGY))),

table(ego1_df$ONTOLOGY))

circos.clear()

circos.initialize(sectors = ego1_df$ID,xlim = c(0,1))

# first GO id

circos.track(sectors = ego1_df$ID,ylim = c(0,1),

bg.col = col,

panel.fun = function(x, y){

circos.text(x = CELL_META$xcenter,y = CELL_META$ycenter,

labels = CELL_META$sector.index,

cex = 0.75)

})

# add count track

circos.track(sectors = ego1_df$ID,ylim = c(0,1),track.height = 0.1,

panel.fun = function(x, y){

# circos.axis(h = "bottom")

})

for (i in 1:nrow(ego1_df)){

circos.rect(xleft = 0,xright = ego1_df$Count[i]/max(ego1_df$Count),

ybottom = 0.25,ytop = 0.75,

sector.index = ego1_df$ID[i],

# track.index = 1,

col = "#9933CC")

# add xaxis

circos.axis(h = "bottom",major.at = c(0,1),labels = c(0,max(ego1_df$Count)),

sector.index = ego1_df$ID[i])

}

# foldchange enriment

circos.track(sectors = ego1_df$ID,ylim = c(0,1),track.height = 0.05)

for (i in 1:nrow(ego1_df)){

circos.text(x = 0.5,y = 0.5,

labels = paste("FC:",round(ego1_df$fc[i],digits = 1),sep = " "),

sector.index = ego1_df$ID[i])

}

circos.track(sectors = ego1_df$ID,ylim = c(0,ceiling(max(ego1_df$fc))))

for (i in 1:nrow(ego1_df)){

circos.barplot(value = ego1_df$fc[i],pos = 0.5,

sector.index = ego1_df$ID[i],

# track.index = 4,

col = "#FF6666")

}

# -log10 pvalue

circos.track(sectors = ego1_df$ID,ylim = c(0,ceiling(max(-log10(ego1_df$pvalue)))))

for (i in 1:nrow(ego1_df)){

circos.barplot(value = -log10(ego1_df$pvalue)[i],pos = 0.5,

sector.index = ego1_df$ID[i],

# track.index = 2,

col = col[i])

}

#

data <- read.table("GO result .txt",header=T,

sep="\t",dec=".",

comment.char="!",na.strings =c("NA"),fill=T,row.names = 1)

#

exp <- read.table("准备文件 (1).txt",header=T,

sep="\t",dec=".",

comment.char="!",na.strings =c("NA"),fill=T,row.names = 1)

#

expr <- exp[,c(1,2)]

#

head(expr)

#

dt <- as_tibble(data[c(3,9)])##

dt$Description <- as.factor(dt$Description)

#

df <- str_split(dt$geneID,",",n=6,simplify=TRUE)

#

dt2 <- data.frame(dt[,1],df)

dt2

#

for (i in 2:7) {

dt2[,i] <- str_replace_all(dt2[,i], pattern="^$", NA_character_)

}

#

dt3 <- pivot_longer(dt2,!Description, names_to = "index",

values_to = "gene",

values_drop_na = TRUE)

dt3

#

n <- nrow(dt3)

dt3$value <- rep(1,n)

#

edge <- dt3[-2]

#

mat <- acast(edge,gene~Description)

#

mat[is.na(mat)] <- 0

#

ind <- row.names(mat)

logFC <- expr[ind,]$logFC

#

mat2 <- data.frame(mat,logFC)

#

mycol <- c("#682487","#547BB4","#4485C7","#7ABBDB","#84BA42","#629C35","#DBB428","#D4562E","#A51C36","#682437")

#

mycol1 <- alpha(mycol,0.6)

#

library(GOplot)

#

GOChord(mat2,space=0.01,

gene.size=5,

process.label=8,

border.size=0.1,

ribbon.col=mycol1,

gene.order='logFC',

lfc.col=c('red','white','blue'))

**Machine learning (Figures 7A-D)**

import pandas as pd

import matplotlib.pyplot as plt

import seaborn as sns

from sklearn.ensemble import RandomForestClassifier

from sklearn.linear_model import Lasso, LassoCV

from sklearn.preprocessing import StandardScaler

import numpy as np

#

plt.rcParams['font.sans-serif'] = [ 'Times New Roman']

plt.rcParams['axes.unicode_minus'] = False #

plt.rcParams['figure.dpi'] = 300 #

#

data = pd.read_excel("箱式图最终数据.xls")

#

X = data.drop("Group", axis=1)

y = data["Group"].map({"Sham": 0, "II/R": 1})

# %% ==================================

rf = RandomForestClassifier(n_estimators=100, random_state=42)

rf.fit(X, y)

rf_importance = pd.DataFrame({

"Gene": X.columns,

"Importance": rf.feature_importances_

}).sort_values("Importance", ascending=False)

#

plt.figure(figsize=(12, 8))

sns.barplot(x="Importance", y="Gene", data=rf_importance.head(20), palette="viridis")

plt.xlabel("Feature Importance Score", fontsize=12)

plt.ylabel("Gene Name", fontsize=12)

plt.tight_layout()

plt.savefig('RF_Feature_Importance.png', dpi=300)

plt.show()

# %% ======================================

#

scaler = StandardScaler()

X_scaled = scaler.fit_transform(X)

# LassoCV

lasso_cv = LassoCV(cv=5, alphas=np.logspace(-4, 0, 100), random_state=42)

lasso_cv.fit(X_scaled, y)

# %% Lasso ==============================

plt.figure(figsize=(12, 8))

alphas = lasso_cv.alphas_ #

#

coefs = []

for alpha in alphas:

lasso = Lasso(alpha=alpha)

lasso.fit(X_scaled, y)

coefs.append(lasso.coef_)

coefs = np.array(coefs)

#

for i in range(coefs.shape[1]):

plt.semilogx(alphas, coefs[:, i], lw=1)

plt.axvline(lasso_cv.alpha_, linestyle='--', color='k', label=f'Optimal alpha: {lasso_cv.alpha_:.3f}')

plt.xlabel("Regularization Strength (Log Scale)", fontsize=12)

plt.ylabel("Standardized Coefficient", fontsize=12)

plt.legend()

plt.savefig('Lasso_Coefficient_Path.png', dpi=300)

plt.show()

#

lasso = Lasso(alpha=lasso_cv.alpha_)

lasso.fit(X_scaled, y)

selected_features = pd.DataFrame({

"Gene": X.columns,

"Coefficient": lasso.coef_

}).query("Coefficient != 0").sort_values("Coefficient", ascending=False)

#

plt.figure(figsize=(12, 6))

sns.barplot(x="Coefficient", y="Gene", data=selected_features,

palette=plt.cm.coolwarm(np.linspace(0, 1, len(selected_features))))

plt.xlabel("Standardized Regression Coefficient", fontsize=12)

plt.ylabel("Gene Name", fontsize=12)

plt.axvline(0, color='gray', linestyle='--')

plt.tight_layout()

plt.savefig('Lasso_Selected_Features.png', dpi=300)

plt.show()

# %% ==============================

#

mse_path = lasso_cv.mse_path_

alphas = lasso_cv.alphas_

best_alpha = lasso_cv.alpha_

# (n_alphas, n_folds)

if mse_path.ndim == 1:

mse_path = mse_path.reshape(-1, 1)

plt.figure(figsize=(12,8))

#

for mse in mse_path.T: #

plt.plot(np.log10(alphas), mse, '-', color='lightgrey', alpha=0.5)

#

mean_mse = np.mean(mse_path, axis=1)

std_mse = np.std(mse_path, axis=1)

plt.plot(np.log10(alphas), mean_mse, 'o-', color='red', label='Mean Binomial Deviance')

plt.fill_between(np.log10(alphas), mean_mse - std_mse, mean_mse + std_mse, color='red', alpha=0.2)

#

plt.axvline(np.log10(best_alpha), linestyle='--', color='k',

label=f'Optimal Log Lambda = {np.log10(best_alpha):.2f}')

plt.xlabel("Log Lambda", fontsize=12)

plt.ylabel("Binomial Deviance", fontsize=12)

plt.legend()

plt.savefig('Lasso_Binomial_Deviance.png', dpi=300)

plt.show()

# %% ========================================

print("【Random Forest Important Features】")

print(rf_importance.head(10).to_string(index=False))

print("\n【Lasso Screening Results】")

print(selected_features.to_string(index=False))

**Construction and verification of the diagnostic model (Figures 8A-C)**

library(rms) #

library(pROC) #

library(rmda) #

library(ggplot2) #

#

train <- read.table("列线图.txt", sep = "\t", row.names = 1,

check.names = F, stringsAsFactors = F, header = T)

#

ddist <- datadist(train)

options(datadist = "ddist")

#

model <- lrm(label ~ Myc+Hif1a+Zfyve1+Sqstm1+Gabarapl1,

data = train, x = TRUE, y = TRUE)

# -------------------- --------------------

nomogram <- nomogram(model,

fun = function(x) 1 / (1 + exp(-x)),

fun.at = c(0.01, 0.05, 0.2, 0.5, 0.9, 0.99),

funlabel = "Risk")

plot(nomogram, cex.axis = 0.8, col.grid = gray(c(0.8, 0.95)))

# -------------------- --------------------

cal_train <- calibrate(model, method = "boot", B = 500)

cal_train <- calibrate(model, method = "crossvalidation", B = 1)

#

plot(cal_train,

subtitles = FALSE,

col = "#2E9FDF", lwd = 2,

xlab = "Predicted Probability",

ylab = "Actual Probability",

main = "Calibration Curve")

# -------------------- --------------------

prob_train <- predict(model, type = "fitted")

roc_train <- roc(response = train$label, predictor = prob_train)

#

auc_value <- auc(roc_train)

#

ggroc(roc_train, legacy.axes = TRUE, color = "#E7B800") +

geom_abline(slope = 1, intercept = 0, linetype = "dashed", color = "gray") +

labs(title = "ROC Curve ",

x = "False Positive Rate",

y = "True Positive Rate") +

theme_minimal() +

theme(plot.title = element_text(hjust = 0.5, face = "bold")) +

annotate("text", x = 0.6, y = 0.2,

label = paste("AUC =", round(auc_value, 3)),

color = "black", size = 5, fontface = "bold")

**Validation of hub genes expression in other datasets (Figure 4C, Figures 9A-C)**

rm(list = ls())

setwd("~/01.小提琴图")

#install.packages('data.table')

library(data.table)

df1 = read.csv('图1.csv')

df1_1 = reshape2::melt(df1,id.vars = 'Group')

head(df1_1)

# df1_1$Group = gsub('II/R','II_R',df1_1$Group)

colnames(df1_1) = c('groups','Gene','value')

gene1 = unique(df1_1$Gene)

da1 = read.csv('All gene1.csv',row.names = 1)

head(da1)

da1_1 = da1[levels(gene1),]

# install.packages('ggpubr')

library(ggpubr)

library(ggplot2)

df1_1$groups = factor(df1_1$groups,levels = unique(df1_1$groups))

p = ggplot(df1_1, aes(x = Gene, y = value, fill = groups)) +

geom_violin(scale = "width", alpha = 0.7, trim =FALSE,width = 0.8,

position = position_dodge(0.9)) +

geom_boxplot(width = 0.15, outlier.shape = NA,

position = position_dodge(0.9),size = .2,

color = "black", alpha = 0.7) +

geom_jitter(position=position_jitterdodge(jitter.height=0,#

jitter.width=0.2,#

dodge.width=0.9),size = .3)+#

scale_fill_manual(values = c('Sham' = "#0C6FAC", 'II/R' = "#D65335")) +

labs(

# title = "Gene Expression Distribution",

x = "",

y = "Gene Expression",

fill = "Group"

) +

theme_bw()+

theme(

axis.text = element_text(angle = 0,color = 'black', hjust = 0.5, size = 10),

legend.position = "right",

)

p

add_signif_stars <- function(p_values) {

case_when(

p_values < 0.0001 ~ "****",

p_values < 0.001 ~ "***",

p_values < 0.01 ~ "**",

p_values < 0.05 ~ "*",

is.na(p_values) ~ NA_character_, #

TRUE ~ ""

)

}

da1_1$sig = add_signif_stars(da1_1$adj.P.Val)

p1 = p+

geom_signif(xmin=.75, xmax=1.25, annotations= da1_1$sig[1], y_position=17,

vjust = 0.05, tip_length = c(.01, .01))+

geom_signif(xmin=1.75, xmax=2.25, annotations= da1_1$sig[2], y_position=17,

vjust = 0.05, tip_length = c(.01, .01))+

geom_signif(xmin=2.75, xmax=3.25, annotations= da1_1$sig[3], y_position=17,

vjust = 0.05, tip_length = c(.01, .01))+

geom_signif(xmin=3.75, xmax=4.25, annotations= da1_1$sig[4], y_position=17,

vjust = 0.05, tip_length = c(.01, .01))+

geom_signif(xmin=4.75, xmax=5.25, annotations= da1_1$sig[5], y_position=17,

vjust = 0.05, tip_length = c(.01, .01))+

geom_signif(xmin=5.75, xmax=6.25, annotations= da1_1$sig[6], y_position=17,

vjust = 0.05, tip_length = c(.01, .01))+

geom_signif(xmin=6.75, xmax=7.25, annotations= da1_1$sig[7], y_position=17,

vjust = 0.05, tip_length = c(.01, .01))+

geom_signif(xmin=7.75, xmax=8.25, annotations= da1_1$sig[8], y_position=17,

vjust = 0.05, tip_length = c(.01, .01))+

geom_signif(xmin=8.75, xmax=9.25, annotations= da1_1$sig[9], y_position=17,

vjust = 0.05, tip_length = c(.01, .01))+

geom_signif(xmin=9.75, xmax=10.25, annotations= da1_1$sig[10], y_position=17,

vjust = 0.05, tip_length = c(.01, .01))+

geom_signif(xmin=10.75, xmax=11.25, annotations= da1_1$sig[11], y_position=17,

vjust = 0.05, tip_length = c(.01, .01))

p1

p1 = p1+ylim(c(4,18))

p1

ggsave('图1.pdf',width = 8,height = 4)

#

rm(list = ls())

setwd("~/01.小提琴图")

#install.packages('data.table')

library(data.table)

df1 = read.csv('图2.CSV',check.names = F)

df1_1 = reshape2::melt(df1,id.vars = 'Group')

head(df1_1)

colnames(df1_1) = c('groups','Gene','value')

gene1 = unique(df1_1$Gene)

library(ggpubr)

library(ggplot2)

df1_1$groups = factor(df1_1$groups,levels = unique(df1_1$groups))

#

library(rstatix)

stat.test <- df1_1 %>%

group_by(Gene) %>%

t_test(value ~ groups) %>%

add_significance("p")

stat.test

write.csv(stat.test,'图2-TTEST.result.csv')

p = ggplot(df1_1, aes(x = Gene, y = value, fill = groups)) +

geom_violin(scale = "width", alpha = 0.7, trim =FALSE,width = 0.8,

position = position_dodge(0.9)) +

geom_boxplot(width = 0.15, outlier.shape = NA,

position = position_dodge(0.9),size = .2,

color = "black", alpha = 0.7) +

geom_jitter(position=position_jitterdodge(jitter.height=0,#

jitter.width=0.2,#

dodge.width=0.9),size = .3)+#

scale_fill_manual(values = c('Sham' = "#0C6FAC", 'II/R' = "#D65335")) +

labs( x = "", y = "Gene Expression" ,fill = "Group") +

theme_bw()+

theme(

axis.text = element_text(angle = 0,color = 'black', hjust = 0.5, size = 10),

legend.position = "right")

p

p1 = p+

geom_signif(xmin=.75, xmax=1.25, annotations= stat.test$p.signif[1], y_position=17,

vjust = 0.05, tip_length = c(.01, .01))+

geom_signif(xmin=1.75, xmax=2.25, annotations= stat.test$p.signif[2], y_position=17,

vjust = 0.05, tip_length = c(.01, .01))+

geom_signif(xmin=2.75, xmax=3.25, annotations= stat.test$p.signif[3], y_position=17,

vjust = 0.05, tip_length = c(.01, .01))+

geom_signif(xmin=3.75, xmax=4.25, annotations= stat.test$p.signif[4], y_position=17,

vjust = 0.05, tip_length = c(.01, .01))+

geom_signif(xmin=4.75, xmax=5.25, annotations= stat.test$p.signif[5], y_position=17,

vjust = 0.05, tip_length = c(.01, .01))

p1

p1 = p1+ylim(c(8,18))

p1

ggsave('图2-TTEST.pdf',width = 7,height = 4)

#

rm(list = ls())

setwd("~/01.小提琴图")

#install.packages('data.table')

library(data.table)

df1 = fread('列线图真 (1).txt',check.names = F) %>% data.frame(row.names = 1)

df1 = df1[c(1:14),]

df1$label = ifelse(df1$label==0,'Sham','II/R')

df1_1 = reshape2::melt(df1,id.vars = 'label')

head(df1_1)

colnames(df1_1) = c('groups','Gene','value')

gene1 = unique(df1_1$Gene)

library(ggpubr)

library(ggplot2)

df1_1$groups = factor(df1_1$groups,levels = unique(df1_1$groups))

# T检验

library(rstatix)

stat.test <- df1_1 %>%

group_by(Gene) %>%

t_test(value ~ groups) %>%

add_significance("p")

stat.test

write.csv(stat.test,'图3-TTEST.result.前7个.csv')

p = ggplot(df1_1, aes(x = Gene, y = value, fill = groups)) +

geom_violin(scale = "width", alpha = 0.7, trim =FALSE,width = 0.8,

position = position_dodge(0.9)) +

geom_boxplot(width = 0.15, outlier.shape = NA,

position = position_dodge(0.9),size = .2,

color = "black", alpha = 0.7) +

geom_jitter(position=position_jitterdodge(jitter.height=0,#

jitter.width=0.2,#

dodge.width=0.9),size = .3)+#

scale_fill_manual(values = c('Sham' = "#0C6FAC", 'II/R' = "#D65335")) +

labs( x = "", y = "Gene Expression" ,fill = "Group") +

theme_bw()+

theme(

axis.text = element_text(angle = 0,color = 'black', hjust = 0.5, size = 10),

legend.position = "right")

p

p1 = p+

geom_signif(xmin=.75, xmax=1.25, annotations= stat.test$p.signif[1], y_position=10.5,

vjust = 0.05, tip_length = c(.01, .01))+

geom_signif(xmin=1.75, xmax=2.25, annotations= stat.test$p.signif[2], y_position=10.5,

vjust = 0.05, tip_length = c(.01, .01))+

geom_signif(xmin=2.75, xmax=3.25, annotations= stat.test$p.signif[3], y_position=10.5,

vjust = 0.05, tip_length = c(.01, .01))+

geom_signif(xmin=3.75, xmax=4.25, annotations= stat.test$p.signif[4], y_position=10.5,

vjust = 0.05, tip_length = c(.01, .01))+

geom_signif(xmin=4.75, xmax=5.25, annotations= stat.test$p.signif[5], y_position=10.5,

vjust = 0.05, tip_length = c(.01, .01))

p1

p1 = p1+ylim(c(6.9,11))

p1

ggsave('图3-列线图真-前7个.pdf',width = 7,height = 4)

#

rm(list = ls())

setwd("~/01.小提琴图")

#install.packages('data.table')

library(data.table)

df1 = fread('列线图真 (1).txt',check.names = F) %>% data.frame(row.names = 1)

df1 = df1[c(1:7,15:21),]

df1$label = ifelse(df1$label==0,'Sham','II/R')

df1_1 = reshape2::melt(df1,id.vars = 'label')

head(df1_1)

colnames(df1_1) = c('groups','Gene','value')

gene1 = unique(df1_1$Gene)

library(ggpubr)

library(ggplot2)

df1_1$groups = factor(df1_1$groups,levels = unique(df1_1$groups))

# T检验

library(rstatix)

stat.test <- df1_1 %>%

group_by(Gene) %>%

t_test(value ~ groups) %>%

add_significance("p")

stat.test

write.csv(stat.test,'图4-TTEST.result.后7个.csv')

p = ggplot(df1_1, aes(x = Gene, y = value, fill = groups)) +

geom_violin(scale = "width", alpha = 0.7, trim =FALSE,width = 0.8,

position = position_dodge(0.9)) +

geom_boxplot(width = 0.15, outlier.shape = NA,

position = position_dodge(0.9),size = .2,

color = "black", alpha = 0.7) +

geom_jitter(position=position_jitterdodge(jitter.height=0,#

jitter.width=0.2,#

dodge.width=0.9),size = .3)+#

scale_fill_manual(values = c('Sham' = "#0C6FAC", 'II/R' = "#D65335")) +

labs( x = "", y = "Gene Expression" ,fill = "Group") +

theme_bw()+

theme(

axis.text = element_text(angle = 0,color = 'black', hjust = 0.5, size = 10),

legend.position = "right")

p

p1 = p+

geom_signif(xmin=.75, xmax=1.25, annotations= stat.test$p.signif[1], y_position=10.5,

vjust = 0.05, tip_length = c(.01, .01))+

geom_signif(xmin=1.75, xmax=2.25, annotations= stat.test$p.signif[2], y_position=10.5,

vjust = 0.05, tip_length = c(.01, .01))+

geom_signif(xmin=2.75, xmax=3.25, annotations= stat.test$p.signif[3], y_position=10.5,

vjust = 0.05, tip_length = c(.01, .01))+

geom_signif(xmin=3.75, xmax=4.25, annotations= stat.test$p.signif[4], y_position=10.5,

vjust = 0.05, tip_length = c(.01, .01))+

geom_signif(xmin=4.75, xmax=5.25, annotations= stat.test$p.signif[5], y_position=10.5,

vjust = 0.05, tip_length = c(.01, .01))

p1

p1 = p1+ylim(c(6.9,11))

p1

ggsave('图3-列线图真-后7个.pdf',width = 7,height = 4)

**Results of immune cell analysis (Figures 10A,10B,10C).**

#

sig_matrix <- read.table("mice.txt", sep = "\t", header = TRUE, row.names = 1)

sig_matrix <- as.matrix(sig_matrix)

#

data_matrix <- read.csv("GSE96733.csv", stringsAsFactors = FALSE)

#

rownames(data_matrix) <- data_matrix$X

data_matrix$X <- NULL

#

data_matrix <- as.matrix(data_matrix)

#

results <- cibersort(sig_matrix = sig_matrix, mixture_file = data_matrix, perm = 100, QN = FALSE)

#

TME.results <- results

TME.cibersort <- as.data.frame(TME.results)

b <- read.table("group.txt",sep = "\t",row.names = 1,check.names = F,header = T)

TME.cibersort <- TME.cibersort[,1:25]

#

non_zero_columns <- colSums(TME.cibersort != 0) > 0

#

TME.cibersort <- TME.cibersort[, non_zero_columns]

identical(rownames(TME.cibersort),rownames(b))

class(b$group)

TME.cibersort$group <- b$group #

TME.cibersort <- TME.cibersort %>% rownames_to_column("sample") #

b <- gather(TME.cibersort,key=CIBERSORT,value = Proportion,-c(group,sample))

#

b <- gather(TME.cibersort, key = "CIBERSORT", value = "Proportion", -c(group, sample))

#

cell_types <- unique(b$CIBERSORT)

#

signif_results <- data.frame(CIBERSORT = character(), p_value = numeric(), label = character(), stringsAsFactors = FALSE)

for (cell in cell_types) {

#

cell_data <- b %>% filter(CIBERSORT == cell)

#

wilcox_res <- wilcox.test(Proportion ~ group, data = cell_data)

#

p_value <- wilcox_res$p.value

#

label <- ifelse(p_value < 0.001, "***",

ifelse(p_value < 0.01, "**",

ifelse(p_value < 0.05, "*", "ns")))

#

signif_results <- rbind(signif_results, data.frame(CIBERSORT = cell, p_value = p_value, label = label, stringsAsFactors = FALSE))

}

#

print(signif_results)

#

b <- merge(b, signif_results, by = "CIBERSORT")

pdf("箱式图.pdf", width = 10, height = 8)

#

ggboxplot(b, x = "CIBERSORT", y = "Proportion", fill = "group", palette = "lancet") +

geom_text(data = signif_results, aes(x = CIBERSORT, y = max(b$Proportion) + 0.05, label = label), inherit.aes = FALSE) +

theme(text = element_text(size = 10), axis.text.x = element_text(angle = 45, hjust = 1))

dev.off()

sig_matrix <- read.table("mice.txt", sep = "\t", header = TRUE, row.names = 1)

sig_matrix <- as.matrix(sig_matrix)

#

data_matrix <- read.csv("GSE96733.csv", stringsAsFactors = FALSE)

#

rownames(data_matrix) <- data_matrix$X

data_matrix$X <- NULL

#

data_matrix <- as.matrix(data_matrix)

#

results <- cibersort(sig_matrix = sig_matrix, mixture_file = data_matrix, perm = 100, QN = FALSE)

results <- as.matrix(results[, 1:(ncol(results) - 3)])

results=rbind(id=colnames(results),results)

save(results,file = "20240424mmdata1.Rdata")

write.table(results, file="CIBERSORT-Results.txt", sep="\t", quote=FALSE, col.names=FALSE)

#

immune <- read.table("CIBERSORT-Results.txt", sep="\t", header=TRUE, check.names=FALSE, row.names=1)

immune[1:5,]

immune_matrix <- as.matrix(immune)

data_transposed <- t(immune_matrix)

#

cellnum <- read.table("CIBERSORT-Results.txt", sep="\t", header=T, row.names=1, check.names=F)

cell.prop <- apply(cellnum, 1, function(x){x/sum(x)})

my36colors <- c('#E5D2DD', '#53A85F', '#F1BB72', '#F3B1A0', '#D6E7A3', '#57C3F3',

'#476D87','#E95C59', '#E59CC4', '#AB3282', '#23452F', '#BD956A', '#8C549C',

'#585658','#9FA3A8', '#E0D4CA', '#5F3D69', '#C5DEBA', '#58A4C3', '#E4C755',

'#F7F398','#AA9A59', '#E63863', '#E39A35', '#C1E6F3', '#6778AE', '#91D0BE',

'#B53E2B', '#712820', '#DCC1DD', '#CCE0F5', '#CCC9E6', '#625D9E', '#68A180',

'#3A6963','#968175')

data4plot <- data.frame()

for (i in 1:ncol(cell.prop)) {

data4plot <- rbind(

data4plot,

cbind(cell.prop[,i],rownames(cell.prop),

rep(colnames(cell.prop)[i],nrow(cell.prop)

)

)

)

}

colnames(data4plot) <- c('proportion','celltype','sample')

data4plot$proportion <- as.numeric(data4plot$proportion)

pdf(file="免疫浸润.pdf",height=10,width=22)

ggplot(data4plot,aes(sample,proportion,fill=celltype))+

geom_bar(stat="identity",position="fill")+

scale_fill_manual(values=my36colors)+# 自定义填充颜色的列表

ggtitle("cell portation")+

theme_bw()+

theme(axis.ticks.length=unit(0.5,'cm'),axis.title.x=element_text(size=1))+

theme(axis.text.x = element_text(angle = 45, hjust = 0.5, vjust = 0.5))+

guides(fill=guide_legend(title=NULL))

dev.off()

#

pdf("免疫相关性图.pdf", height=13, width=13)

par(oma=c(0.5, 1, 1, 1.2))

immune <- immune[, colMeans(immune) > 0]

M <- cor(immune)

corrplot(M,

order="hclust",

method="color",

addCoef.col="black",

diag=TRUE,

tl.col="black",

col=colorRampPalette(c("blue", "white", "red"))(50))

dev.off()

#######gene——cell########

#

library(ggplot2)

library(reshape2)

#

gene_expression <- read.table("GSE96733genes.txt", header = TRUE, row.names = 1)

cell_types <- read.table("CIBERSORT-Results.txt", header = TRUE, row.names = 1)

#

gene_expression <- as.data.frame(t(gene_expression))

cell_types <- as.data.frame(t(cell_types))

cell_types <- as.data.frame(t(cell_types))

#

cell_types <- cell_types[, colSums(cell_types != 0) > 0]

#

results <- data.frame(

Gene = character(),

CellType = character(),

Correlation = numeric(),

PValue = numeric(),

stringsAsFactors = FALSE

)

#

for (gene in colnames(gene_expression)) {

for (cell_type in colnames(cell_types)) {

cor_test <- cor.test(

gene_expression[[gene]],

cell_types[[cell_type]],

method = "spearman"

)

results <- rbind(

results,

data.frame(

Gene = gene,

CellType = cell_type,

Correlation = cor_test$estimate,

PValue = cor_test$p.value

)

)

}

}

#

results$Significance <- ifelse(results$PValue < 0.001, "***",

ifelse(results$PValue < 0.01, "**",

ifelse(results$PValue < 0.05, "*", "")))

#

heatmap_data <- dcast(results, Gene ~ CellType, value.var = "Correlation")

pdf('gene_cell相关性.pdf',width = 8,height = 5)

#

ggplot(results, aes(x = CellType, y = Gene)) +

geom_tile(aes(fill = Correlation), color = "white") +

scale_fill_gradient2(low = "blue", high = "red", mid = "white", midpoint = 0) +

geom_text(aes(label = Significance), color = "black", size = 3) +

theme_minimal() +

theme(

axis.text.x = element_text(angle = 45, hjust = 1),

axis.text.y = element_text(size = 8),

axis.title = element_blank()

) +

labs(fill = "Spearman Correlation")

dev.off()

**Results of drugs prediction(Figure 12).**

#

library(ggplot2)

enrichment_results <- 药物

enrichment_results[,1] <- -log10(enrichment_results$Enrichment.FDR)

p <- ggplot(enrichment_results, aes(x = Pathway, y = Fold.Enrichment)) +

geom_point(aes(size = nGenes, color = Enrichment.FDR)) +

geom_segment(aes(xend = Pathway, yend = 0, color = Enrichment.FDR), size = 1) +

coord_flip() +

scale_color_gradient(name = "−log10(FDR)", low = "blue", high = "red") +

theme_minimal() +

theme(panel.background = element_rect(fill = "#D3D3D3", colour = NA),

panel.grid.major = element_blank(),

panel.grid.minor = element_blank(),

axis.text.x = element_text(angle = 45, hjust = 1, family = "Times"),

legend.position = "right",

axis.line = element_blank(),

axis.ticks = element_line(linetype = "dashed"),

axis.title = element_text(family = "Times", size = 13, vjust = 0),

axis.text = element_text(family = "Times", size = 11, colour = "black", hjust = 0),

plot.title = element_text(family = "Times", size = 13)) +

labs(title = NULL,

x = NULL,

y = "Fold Enrichment") +

guides(size = guide_legend(title = "N. of Genes"))

#

print(p)
